# Low radiotherapy dose is suitable for brain metastases in SCLC compared with high dose

**DOI:** 10.3389/fonc.2023.1245506

**Published:** 2023-09-15

**Authors:** Liming Xu, Kunning Zhang, Haonan Han, Han Sun, Yajing Yuan, Jun Wang, Lujun Zhao, Ping Wang

**Affiliations:** ^1^ Department of Radiation Oncology, Tianjin Medical University Cancer Institute and Hospital, National Clinical Research Center for Cancer, Key Laboratory of Cancer Prevention and Therapy, Tianjin’s Clinical Research Center for Cancer, Tianjin, China; ^2^ Hubei Key Laboratory of Tumor Microenvironment and Immunotherapy, College of Basic Medical Sciences, China Three Gorges University, Yichang, China; ^3^ Department of Radiation Oncology, Cancer Center/National Clinical Research, Center for Cancer/Cancer Hospital & Shenzhen Hospital, Chinese Academy of Medical Sciences and Peking Union Medical College, Shenzhen, China; ^4^ Department of Anesthesia, Tianjin Medical University Cancer Institute and Hospital, National Clinical Research Center for Cancer, Key Laboratory of Cancer Prevention and Therapy, Tianjin’s Clinical Research Center for Cancer, Tianjin, China; ^5^ Department of Radiation Oncology, Tianjin Cancer Hospital Airport Hospital, Tianjin, China

**Keywords:** carcinoma, brain metastases, small cell lung cancer, radiotherapy, prognosis

## Abstract

**Objective:**

This study was designed to evaluate the suitable radiotherapy dose in SCLC patients with BM.

**Methods:**

A retrospective analysis was performed among 121 patients on the prognosis of BM of SCLC who were admitted to our hospital from 2013 to 2023. They all received first line chemotherapy. 80 patients of them received TRT after chemotherapy. The Chi square method was used to compare the categorical data. Univariate survival analysis was estimated by Kaplan Meier method and the logrank was used to compare survival curves between groups. A multivariate prognostic analysis was made by the Cox proportional hazard model. The iOS and iLC of two groups of low dose and high dose were analyzed after propensity score matching (PSM).

**Results:**

In all the patients, the median follow-up time was 18.6 months (range 6.30~85.7), the 2-year iOS and iLC rates were 15.4% and 70.3%, respectively, and cerebral necrosis occurred in 2 patients. In univariate analysis related to iOS, extracranial disease control (p=0.023), higher DS-GPA (≥2) (p=0.016), immunotherapy (p=0.049), low-dose(p=0.030), and WBRT+SIB (p=0.009) were significantly associated with an increase in survival rate. After PSM, the 2-year iOS of low dose (n=49) was significantly higher than that of high dose (n=49) (P=0.025), while the 2-year iLC was not significantly improved (P=0.267). In DS-GPA < 2 subgroup, the iOS of low dose group was significantly higher than that of high dose group (p=0.019). In the DS-GPA ≥ 2 subgroup, the 2-year iLC of the low dose group was significantly inferior than that of the high dose group (p=0.044).

**Conclusions:**

The iLC was improved along with increasing radiotherapy dose, but high dose had inferior iOS compared to low dose, while there were not significantly improving iLC when radiotherapy BED >56Gy. But in patients with DS-GPA≥2 subgroup, high dose brought better iLC benefits.

## Introduction

Lung cancer is the second most common cancer worldwide. It is the most common cancer in men and the second most common cancer in women. There were more than 2.2 million new cases of lung cancer in 2020 (1). Small cell lung cancer (SCLC) is a high-grade neuroendocrine tumor characterized by rapid growth, early metastatic spread, and initial responsiveness to therapy. It represents about 15% of all lung cancers. Approximately 18% of the patients were found to have BM at the time of diagnosis. In approximately 33% of the cases, these BM did not cause symptoms. More than 50% will develop BM within 2 years (2). BM were found to have a negative effect on survival in patients with SCLC. The median survival time after BM was 8.7 months and 3-year OS rate was 15.0%, the median survival time of patients without BM was 20.1 months and 3-year OS was 33.4% (P=0.014) (3, 4). Patients with BM were subsequently treated with palliative therapy. The standard treatment for SCLC BM is still WBRT, with an overall effective rate (ORR) of approximately 50% -80% (5). Magnetic resonance imaging (MRI) is a more sensitive technique to detect BM, In the MRI era, the estimated prevalence of BM increased by 14% (6). Patients with asymptomatic BM by MRI were more detected, and had a better prognosis (7). In our previous clinical studies, we have found that WBRT combined with radiation boost can improve the overall survival (OS) of SCLC patients with BM (8). However, the suitable radiotherapy dose of BM and efficacy are not very clear. This article analyzes the efficacy and safety of low dose or high dose in SCLC patients with BM retrospectively, in order to optimize WBRT+ suitable radiation boost dose for SCLC patients with BM.

## Materials and methods

### Inclusion criteria

We retrospectively analyzed the clinical characteristics of SCLC BM patients who received brain radiation therapy from 2013 to 2023. All patients received treatment from Tianjin Cancer Institute and Hospital. This study was approved by the ethics committee of Tianjin Medical University Cancer Hospital. This research on patient services in our hospital was an analysis of patients’ medical data, which did not involve human experiments or compensation. The Tianjin Medical University Cancer Hospital approved the study data collection from the hospital information system. It is typically diagnosed in small biopsies or cytology specimens, demonstrating neuroendocrine features of SCLC (9). All clinical data of patients are from outpatient or inpatient clinical records. The patient underwent standardized physical examinations, including CT scans of the neck, chest, and abdomen, brain MRI, as well as ECT or PET/CT. ES-SCLC was defined in this study depending on the staging system of the Veterans Administration Lung Study Group, (VALG).

### Initial treatment strategy

All patients underwent chemotherapy and/or combined concurrent radiotherapy/sequential TRT. Chemotherapy strategies: The etoposide(100 mg from days 1 to 5) with either cisplatin (30 mg/m from days 1 to 3) or carboplatin (500 mg for day 1) (platinum–etoposide) as the first-line chemotherapy regimen. The median chemotherapy cycles are 6 (range 2-6). TRT strategies: The tumor and metastatic lymph nodes were defined as the GTV. The CTV encompassed the tumor bed after chemotherapy, and the draining area of metastatic lymph nodes before chemotherapy, which was expanded from the GTV by a 5 mm uniform margin. The PTV was evenly extended 0.5 to 1cm uniform margin on the basis of CTV. The prescription dose was 50-63Gy in 25-30 fractions, 1.8-2.1 Gy per fraction at one fraction per day. All patients were treated WBRT with radiation boost by IMRT or SRS. The WBRT plus a radiation boost strategy: WBRT was performed with 6 MV photon beams using opposed lateral fields (90° and 270°) with a total dose of 30 Gy (3 Gy per fraction administered in 10 fractions at one fraction per day). The SRS was administered using a Cyberknife (Accuracy, Sunnyvale,California, USA) or X-knife after the WBRT in 56 patients. The GTV encompassed contrast-enhancing tumor on MRI and were reviewed by the radiation oncologist and the neurosurgeon based on the tumor volume, tumor location, and neurological symptoms. The PTV was defined as the 1 to 2 mm margin to the GTV. The administered radiation dose was 8.5-19 Gy in 1-3fractions with 6.3-18.0 Gy per fraction and one fraction per day(BED=10.3-29.9Gy). The IMRT simultaneous integrated boost WBRT (WBRT-SIB) was administered in 65 patients. The GTV was contoured based on the tumor from contrast-enhanced MRI scans. The PTV of brain metastases (PTVbm) was defined as the 3mm margin to the GTV with the dose of 35-50 Gy in 10 fractions with 3.5-5 Gy per fraction and one fraction per day. In general, we treated BMs less than 10 mm in maximum diameter with a prescription of 50 Gy(BED=75Gy); BMs larger than 10 mm but smaller than 30 mm with 40 Gy(BED=56Gy); and BMs larger than 30mm and less than 40 mm with 35Gy(BED=47.25Gy). The prescription of dose fractionation was consistent with previous clinical trials (10, 11). The PTV was expanded from the contour of the brain by the 3mm uniform margin with the dose of 30 Gy in 10 fractions with 3 Gy per fraction and one fraction per day.

### Efficacy evaluation, follow-up and side effects

Acute toxicity reactions are classified according to CTCAE version 5.0, and late toxicity is classified according to RTOG standards. Evaluate the efficacy of solid tumors according to RECIST 1.1. Repeat the baseline assessment every two cycles and every 6-8 weeks after treatment interruption until the disease progresses. Intracranial overall survival (iOS)is defined as the period from the start of BM diagnosis by imaging (MRI or enhanced CT) until the event occurs or the last follow-up. Intracranial local control survival (iLC) is defined as the time from the start of BM diagnosis by imaging (MRI or enhanced CT) until the first event of intracranial local failure.

### Statistical analysis

All survival analyses were conducted using the Kaplan Meier method. Compare survival curves between different groups using logarithmic rank test and use χ^2^ test and compare classified data. Cox proportional hazard regression model was used for Multivariate analysis of survival rate. Two groups of patients were subjected in a 1:1 ratio by PSM to analyze and control the confounding variables, including diagnosis-specific Graded Prognostic Assessment (DS-GPA) score, number of intracranial metastases, maximum diameter of metastases, and progression of extracranial diseases. In this study, the p-values were all one-way tests, and there was a statistically significant difference between groups when p<0.05. All analyses were conducted using SPSS software version 25.0.

## Result

### Clinical features

The patient characteristics of 121 patients were shown in **Table 1**. The majority of patients are male (n=100, 82.6%). The median age is 61 years (range 18-83 years). Most patients have severe smoking (smoking index ≥ 400, n=90,74.4%). Most patients have a superior Karnofsky performance status (KPS) score (KPS score ≥ 80, n=107, 88.4). The most common metastatic organs are as follows: 40 cases (33.1%) had bone metastasis; 21 cases (17.4%) had distant lymph node metastasis; 20 cases (16.5%) had lung metastasis; 15 cases (12.4%) had pleural metastasis; 14 cases (11.6%) had adrenal metastasis; Liver metastasis occurred in 12 cases (10.0%). Most patients received more than 4 cycles of chemotherapy (n=115, 95.0%). 86 patients (71.1%) responded to chemotherapy. Only 14 patients received immunotherapy (immune checkpoint inhibitors, ICIs), and 5 patients received treatment with arotinib.

**Table 1:** Distribution of the 121 patients’ treatment and clinical characteristics.

Characteristic	Number	Ratio (%)
Age (yrs)		
** <65 yrs**	86	71.1
** ≥65 yrs**	35	28.9
Gender		
** male**	100	82.6
** female**	21	17.4
Smoke index		
** ≥400**	90	74.4
** <400**	31	25.6
Family history of tumors		
** No**	97	80.2
** Yes**	24	19.8
Weight loss		
** >5%**	96	79.3
** ≤5%**	25	20.7
KPS		
** ≥80**	107	88.4
** <80**	14	11.6
**Thoracic radiation therapy dose**		
** <50Gy**	12	9.9
** ≥50Gy**	68	56.2
Stage		
** LS-SCLC**	67	55.4
** ES-SCLC**	54	44.6
Number of BMs		
** 1**	51	42.1
** 2-3**	62	51.2
** >3**	8	6.6
Maximum diameter of the largest tumor(cm)		
** ≤ 2.0**	76	62.8
** > 2.0**	45	37.2
Interval from diagnosis of SCLC to BMs (mths)		
** ≤ 10**	63	52.1
** > 10**	58	47.9
Extracranial disease control status		
** Yes**	40	33.1
** No**	81	66.9
**(Diagnosis-specific Graded Prognostic Assessment) DS-GPA**		
<2	31	25.6
≥2	90	74.4
Immunotherapy (ICI)		
** Yes**	14	11.6
** No**	107	88.4
Targeted therapy (anti-angiogenic therapy)		
** Yes**	5	4.1
** No**	116	95.9
Brain radiotherapy		
** WBRT+SIB**	66	54.5
** WBRT+SBRT**	55	45.5
Radiotherapy dose (BED)		
** low-dose (BED ≤ 56Gy)**	**65**	**48.1**
** high-dose (BED>56Gy)**	**56**	**51.9**

### Survival and side effects

**Figure 1:**
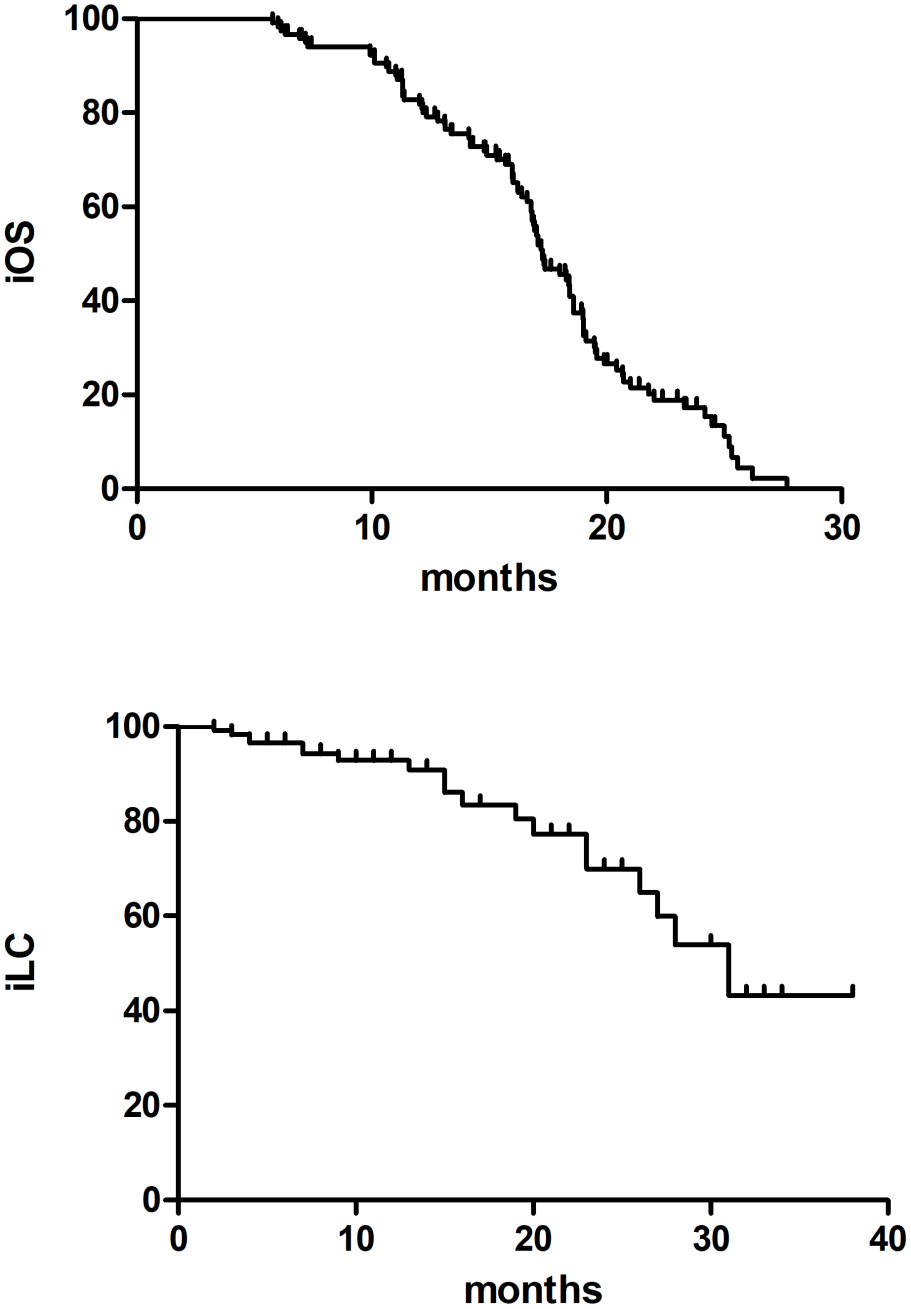
The iOS and iLC in 121 SCLC BM patients.

The median follow-up time was 18.6 months (ranging from 6.30 to 85.7months) with 2 patients lost to follow-up. The 2-year incidence of iOS and iLC was 15.4% and 70.3%, respectively (**Figure 1**). 92 patients died of disease progression, 1 patient died of radiation pneumonia, and 2 patients developed radiation brain necrosis. A few of patients have experienced treatment related toxic side effects, mainly including nausea, vomiting, dizziness, headache, leukopenia, radiation brain necrosis, etc. (The side effects of the low dose group and the high dose group are shown in **Table 2**).

**Table 2:** Adverse reactions of 98 patients of SCLC with BMs in t in two treatment groups after PSM matching.

Adverse reactions	Low dose (49)		High dose (49)		P value
	1 or 2 (%)	3 (%)	1 or 2 (%)	3 (%)	
**Weakness**	**8**	**3**	**8**	**2**	**0.550**
**Headache**	**15**	**10**	**19**	**9**	**0.379**
**Dizziness**	**12**	**8**	**10**	**8**	**0.520**
**Nausea**	**20**	**7**	**19**	**7**	**0.483**
**Vomit**	**4**	**0**	**3**	**0**	
**Fever**	**1**	**0**	**0**	**0**	
**Leukopenia**	**20**	**5**	**18**	**5**	**0.581**
**Thrombocytopenia**	**2**	**0**	**2**	**1**	
**Radiation dermatitis**	**2**	**0**	**2**	**0**	**0.167**
**Disorders of consciousness**	**0**	**0**	**0**	**0**	
**Radiation brain necrosis**	**1**	**0**	**0**	**1**	**0.500**

Assess the predictive significance of patient and disease characteristics for iOS. Due to the small number (only 5) patients who were treated with arotinib, this factor is not suitable for analysis. In univariate analysis related to iOS, extracranial disease control (p=0.023), higher DS-GPA (≥2) (p=0.016), immunotherapy (p=0.049), low dose (p=0.030), and WBRT+SIB (p=0.009) were significantly associated with an increase in survival rate (**Table 3**). Age, gender, weight loss, smoking history, TRT dosage, and the time interval for BM after diagnosis were not significantly observed in staging and the number of brain metastases (all factors p>0.05). Multivariate covariates analysis of factors related to iOS were further analyzed with p<0.05 in univariate Cox regression model analysis. The extracranial progress control (p=0.049) and higher DS-GPA (≥2) (p=0.014) can significantly improve iOS by multivariate analysis (**Table 3**). However, there was no significant difference in immunotherapy, BM radiotherapy strategy and radiotherapy dose (p>0.05 for all factors).

**Table 3:** 121 patients’ clinical and treatment characteristics and survival-related factors on iOS in univariate and multivariate analysis.

Characteristic	univariate analysis				multivariate analysis			
	HR	95% CI		P value	HR	95% CI		P value
**Age (<65 yrs v.s.≥65 yrs)**	**0.580**	**0.361**	**0.933**	**0.055**				
**Gender(male v.s.female)**	**0.951**	**.526**	**1.721**	**0.869**				
**Smoke index (≥400v.s. <400)**	**0.872**	**0.536**	**1.419**	**0.582**				
**Family history of tumors (no v.s.yes)**	**1.310**	**0.786**	**2.182**	**0.301**				
**Weight loss (>5%v.s.≤5%)**	**0.939**	**0.542**	**1.628**	**0.824**				
**Karnofsky scoring (≥80v.s. <80)**	**0.807**	**0.427**	**1.526**	**0.510**				
**Thoracic radiation therapy dose (≥50Gyv.s.<50Gy)**	**0.919**	**0.450**	**1.875**	**0.816**				
**Stage [LS (limited stage) vs. ES (extensive stage)]**	**1.301**	**0.851**	**1.990**	**0.224**				
**Number of BMs (1v.s.2-3v.s.>3)**	**1.159**	**0.802**	**1.675**	**0.432**				
**Maximum diameter of the largest tumor (cm) (≤ 2.0v.s.> 2.0)**	**0.976**	**0.636**	**1.498**	**0.912**				
**Interval from diagnosis of SCLC to BMs (mths) (≤ 9v.s> 9)**	**0.878**	**0.576**	**1.338**	**0.545**				
**Extracranial disease control status (yes v.s. no)**	**1.723**	**1.077**	**2.756**	**0.023**	**1.628**	**1.001**	**2.648**	**0.049**
**Diagnosis-specific Graded Prognostic Assessment (DS-GPA) (<2 v.s.≥2)**	**0.522**	**0.307**	**0.886**	**0.016**	**0.508**	**0.296**	**0.873**	**0.014**
**Immunotherapy (no v.s.yes )**	**0.444**	**0.193**	**1.020**	**0.049**	**0.492**	**0.206**	**1.175**	**0.110**
**brain metastasis (BM) Radiotherapy [whole brain radiotherapy (WBRT)+simultaneous integrated boost (SIB) v.s.WBRT+stereotactic radiosurgery (SRS)]**	**0.563**	**0.367**	**0.863**	**0.009**	**0.268**	**0.035**	**2.075**	**0.207**
**Radiotherapy dose (low-dose v.s.high dose)**	**1.607**	**1.048**	**2.465**	**0.030**	**0.438**	**0.056**	**3.407**	**0.430**

### Comparison of survival between the low dose group and the low dose group

**Figure 2:**
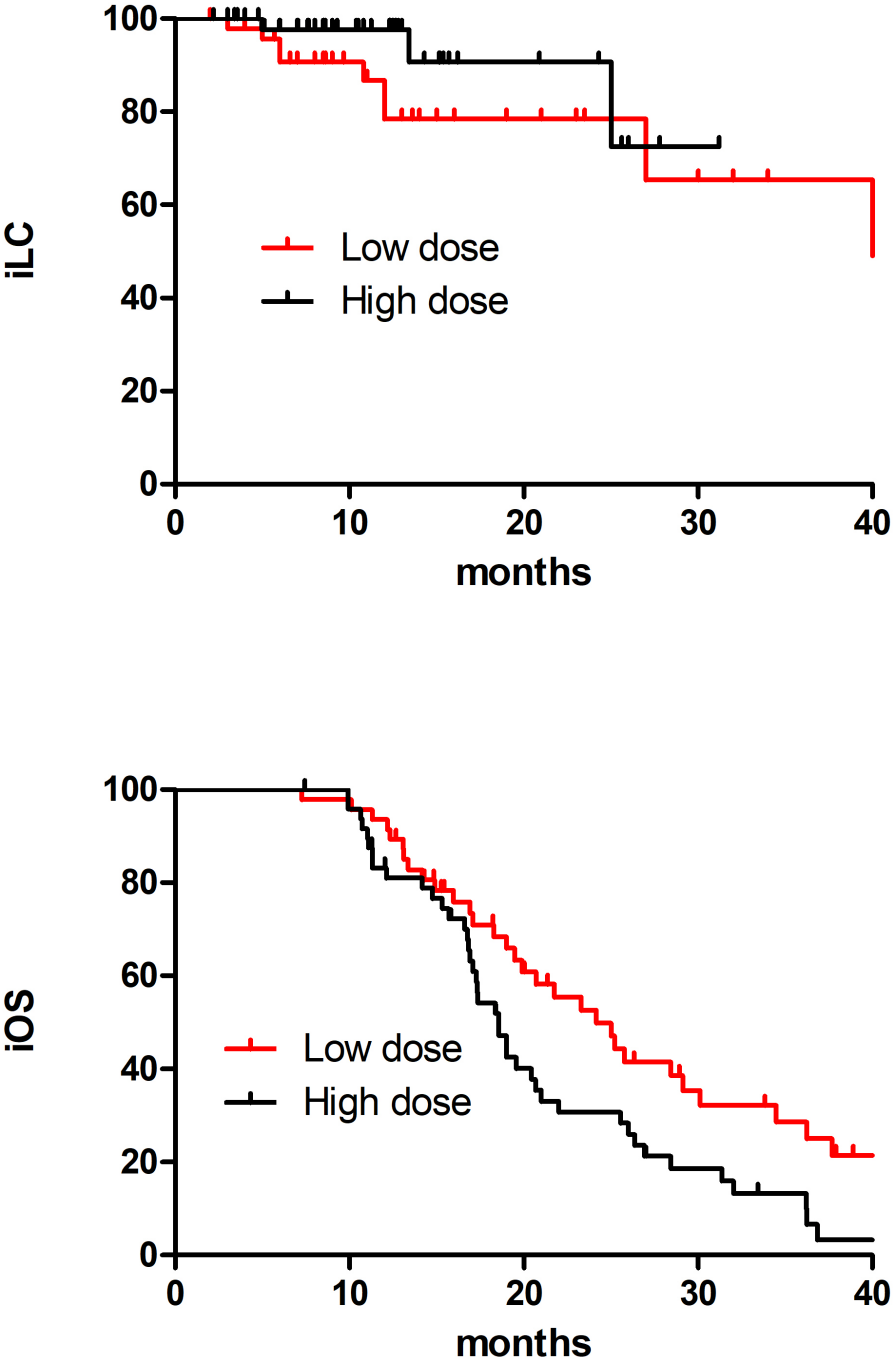
The iOS and iLC in 96 SCLC BM patients in low dose and high dose after PSM matching.

This study divided 121 patients into two groups according to BM radiotherapy, with 65 receiving low dose treatment and 56 receiving high dose. As shown in **Table 4**, compared to the low dose group, the high dose group had more patients with more weight loss >5%(9.2%vs33.9%, p=0.001), a smaller maximum diameter of BMs (53.8%vs73.2%, p=0.022), longer interval from diagnosis of SCLC to BMs (36.9% vs69.6%, p=0.000)and SRS (1.5%vs96.4%, p=0.000), which resulted in an imbalance between the two groups, and there was no significant difference in other baseline characteristics between the two treatment groups. Because the difference between SIB and SRS was too significant, and the majority of the low dose group had SIB and the majority of high dose group had SBRT, so the brain radiotherapy strategy factor was excluded. After a 1:1 PSM analysis, the baseline characteristics of the two groups of patients were well balanced (**Table 5**). The low dose group (n=49) was significantly superior than that of the high dose group (n=49) about the 2-year iOS (47.1% vs 30.7%, P=0.025), while there was no increasing significantly about the 2-year iLC in high dose(65.3% vs 91.9%, P=0.267) (**Figure 2**).

**Table 4:** Distribution of the 121 patients’ treatment and clinical characteristics in two treatment groups.

Characteristic	Low dose (n=65) (%)	High dose (n=56)(%)	P value
**Age (<65 yrs)**	**67.7%**	**75.0%**	**0.377**
**Gender(male)**	**84.6%**	**80.4%**	**0.537**
**Smoke index≥400**	**83.1%**	**74.5%**	**0.189**
**Family history of tumors (yes)**	**24.6%**	**14.3%**	**0.116**
**Weight loss >5%(yes)**	**9.2%**	**33.9%**	**0.001**
**Karnofsky scoring≥80**	**86.2%**	**91.1%**	**0.399**
**Thoracic radiation therapy dose ≥50Gy**	**79.2%**	**87.5%**	**0.263**
**Stage [limited stagesmall cell lung cancer (LS-SCLC)]**	**50.8%**	**60.7%**	**0.181**
**Number of brain metastasis (BMs)**			**0.306**
**1**	**44.6%**	**39.3%**	
**2-3**	**46.2%**	**57.1%**	
**>3**	**9.2%**	**3.6%**	
**Diagnosis-specific Graded Prognostic Assessment (DS-GPA) (<2)**	**32.3%**	**17.9%**	**0.053**
**Maximum diameter of the largest tumor(≤2.0cm)**	**53.8%**	**73.2%**	**0.022**
**Interval from diagnosis of SCLC to BMs (>10 mths)**	**36.9%**	**69.6%**	**0.000**
**Extracranial disease control status (yes)**	**61.5%**	**73.2%**	**0.173**
**whole brain radiotherapy (WBRT)+stereotactic radiosurgery (SRS)**	**1.5%**	**96.4%**	**0.000**

**Table 5:** Distribution of the 98 patient treatment and clinical characteristics in two treatment groups after PSM matching.

Characteristic	low dose(%)	high dose(%)	P value
**Age (<65 yrs)**	**71.4%**	**79.6%**	**0.241**
**Gender(male)**	**87.8%**	**77.6%**	**0.143**
**Smoke index≥400**	**80.0%**	**70.8%**	**0.217**
**Family history of tumors (yes)**	**24.5%**	**12.2%**	**0.096**
**Weight loss >5%(no)**	**18.4%**	**28.6%**	**0.170**
**KPS≥80**	**83.7%**	**91.8%**	**0.187**
**TRT dose ≥50Gy**	**77.3%**	**85.4%**	**0.302**
**Stage (LS-SCLC)**	**51.0%**	**67.3%**	**0.075**
**Number of BMs**			**0.206**
**1**	**49.0%**	**40.8%**	
**2-3**	**42.9%**	**57.1%**	
**>3**	**8.2%**	**2.0%**	
**GPA (<2)**	**30.6%**	**16.3%**	**0.176**
**Maximum diameter of the largest tumor(≤2.0cm)**	**59.2%**	**71.4%**	**0.144**
**Interval from diagnosis of SCLC to BMs (≤10 mths)**	**51.0%**	**65.3%**	**0.110**
**Extracranial disease control status (yes)**	**77.6%**	**83.7%**	**0.305**

**Figure 3:**
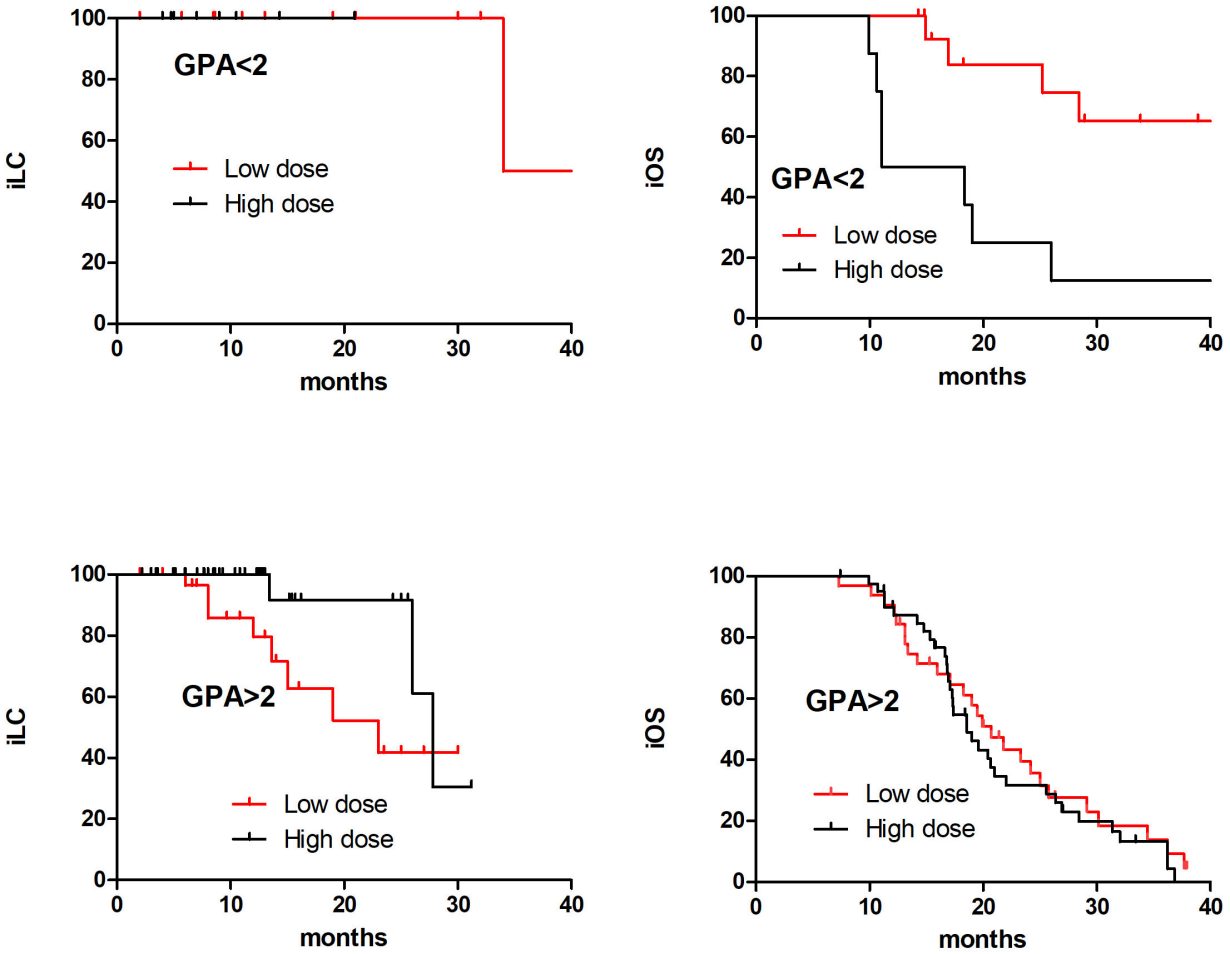
The iOS and iLC in 98 SCLC BM patients with different DS-GPA scores in low dose and high dose after PSM matching.

Further analysis was conducted on the prognosis of the low and high dose groups in different DS-GPA scores. It was found that in the GPA<2 subgroup, the 2-year iOS in the low dose group was significantly superior than that in the high dose group (65.3% and 25.0%, respectively, at, p=0.019), while in the DS-GPA ≥ 2 subgroup, there was no significant difference between the low dose group and high dose group (31.6% and 28.8%, respectively, p=0.502); in the DS-GPA<2 subgroup, there was no significant difference between the low dose group and high dose group in the 2-year iLC (100% and 100%, respectively). In the DS-GPA ≥ 2 subgroup, the 2-year iLC of the low dose group was significantly inferior than that of the high dose group (52.2% and 91.7%, respectively, p=0.044) (**Figure 3**).

## Discussion

In this study, we conducted a further study about radiotherapy dose in SCLC patients with BMs. Our previous studies have confirmed that WBRT with additional radiation boost is more effective than the WBRT alone group in prolonging the survival of SCLC patients with BMs (12). On this basis, we further investigate the different radiotherapy dose in brain metastases: low dose and high dose, which have effects on iOS, iLC, and radiotherapy side effects. The results showed that there was no significant difference in the side effects of different radiotherapy dose (**Table 2**). After PSM matching, the 2-year iOS of the low dose group was significantly superior than that of the high dose group. Further analysis revealed that in the DS-GPA<2 subgroup, the iOS in the low dose group was significantly superior than that in the high dose group; In subgroups of DS-GPA ≥ 2, the iLC in the high dose group was significantly superior than that in the low dose group.

More than 50% of SCLC patients may have BMs during the disease developing, and the prognosis is poor after the occurrence of BM. The treatment of BM in SCLC patients is different from other solid tumors because SCLC is a very aggressive, poorly differentiated, and high-grade neuroendocrine carcinoma (13). Even in patients with stage I-III SCLC who received surgical resection, the cumulative incidence of brain metastases was as high as 30% (14). According to the time of BMs to initial diagnosis, clinical manifestations of BMs (synchronous and asynchronous BM), BM treatment plans are different. In addition, the radiotherapy strategy also considers the patient’s extracranial disease control status. Considering the high frequency of intracranial recurrence, SRS or surgical treatment SRS for limited BM from SCLC is not a standard of care. However, more evidence suggests that SBRT alone is feasible for treating BM in SCLC patients. However, in a large multicenter analysis, it was found that compared to SRS alone, WBRT improved TTP (HR 0.38, p<0.001), but did not significantly improve OS (median OS, 6.5 [SRS] vs 5.2 months [WBRT], p=0.003) (15).

Several clinical studies have reported the role of WBRT plus boost radiotherapy in the treatment of BM. Andrews et al (16) recruited 331 patients with 1-3 BMs, and found that WBRT combined with SRS significantly improved the 1-year local control of intracranial metastasis (82% vs 71%, p=0.013) compared with WBRT alone. In addition, compared to WBRT alone, WBRT+SRS improved the survival of patients with single BM, with a median OS of 4.9 months and 6.5 months, respectively (p=0.039) (15). The recent report showed that WBRT+SRS significantly improved the OS in the DS-GPA 2.5-4.0 subgroup, with median OS of 16.7 months and 10.6 months, respectively (p=0.04) (15, 17, 18).

In previous studies, some prognostic factors such as KPS, age, extracranial disease control status, and number of BM were identified in SCLC patients with BM. In this study, by univariate and multivariate analysis, extracranial disease control, and the higher DS-GPA were significantly related to the superior of OS. In addition, although the number of immunotherapy cases is relatively small, OS is still significantly affected by immunotherapy in univariate analysis.

With the promotion of SCLC comprehensive treatment and the application of immunotherapy, the OS of SCLC patients has significantly improved, reaching over 12 months. Therefore, the radiotherapy strategies for BM need further study. A meta-analysis showed that for patients with BM receiving SRS, when the BED was 40, 50, and 60Gy, 1-year iLC were 73%, 78%, and 84%, respectively, and 2-year iLC were 62%, 69%, and 81%, respectively (19). The iLC was improved along with increasing radiotherapy dose. A multi-center retrospective study reported that BED dose >50.7Gy was associated with improved OS in patients with BM (23.3 months vs. 8.2 months, p < 0.01) (20). Another retrospective study suggested that the BED >47.4Gy brain radiotherapy can improve OS and iPFS (21). This study mainly compared the impact on prognosis with different radiotherapy dose, and the results showed that high dose had inferior iOS compared to low dose, while there were not significantly improving in iLC when BED >56Gy. This may be related to the low GPA score, large BM, multiple BM in the high group (22–24). However, there was no significant difference in clinical characteristic distribution after PSM in this study, indicating that low dose had a survival advantage for BM patients. Based on the classification of DS-GPA, we further analyzed the prognosis of different DS-GPA scores in the low and high dose groups. We found that in the GPA<2 subgroup, the iOS in the low dose group was significantly superior than that in the high dose group; in subgroups with GPA ≥ 2, the iLC in the high dose group was significantly superior than that in the low dose group. This inconsistent phenomenon suggested that the role of chemotherapy and immunity may be more important for SCLC patients with BM in the GPA<2 subgroup.

This study has the following limitations. Firstly, this study is a retrospective analysis, and the distribution of clinical features is not very uniform. Secondly, this is a small sample retrospective study with choice bias, which should be verified by further prospective cohort study.

## Conclusions

To our knowledge, this is the first retrospective study to evaluate WBRT with different radiotherapy boost approaches (SIB and SRS) in SCLC patients with BM. Our study found that the iLC was improved along with increasing radiotherapy dose, but high dose had inferior iOS compared to low dose, while there were not significantly improving iLC when BED >56Gy. In patients with GPA**≥**2 subgroup, high dose brought better iLC benefits. This surprising result suggested that the iLC was not improved iOS along with increasing radiotherapy dose when the radiotherapy dose reached to a certain extent, which needed further observed.

## Data availability statement

The original contributions presented in the study are included in the article/supplementary material. Further inquiries can be directed to the corresponding authors.

## Ethics statement

The studies involving humans were approved by The ethics committee of Tianjin Medical University Cancer Hospital. The studies were conducted in accordance with the local legislation and institutional requirements. Written informed consent for participation was not required from the participants or the participants’ legal guardians/next of kin in accordance with the national legislation and institutional requirements.

## Author contributions

LX, KZ and HH performed data acquisition, the statistical analysis and drafted the manuscript, the three authors contributed equally to the study. HS and YY performed data acquisition and the statistical analysis. JW, LZ and PW critically reviewed the manuscript. All authors contributed to the article and approved the submitted version.
